# The “Iron Triangle” of Healthcare Services: Evaluating an Integrated Healthcare Alliance’s Impact on Primary Care in Shenzhen, China

**DOI:** 10.5334/ijic.10192

**Published:** 2026-09-30

**Authors:** Shilan Tang, Miao Yu, Xizi Wan

**Affiliations:** 1 General Management Department, Shenzhen Dapeng New District Medical and Health Group, Shenzhen, China; 2 School of Healthcare Management, Tsinghua Medicine, Tsinghua University, Beijing, China; 3 Tsinghua Shenzhen International Graduate School, Tsinghua University, Shenzhen, China

**Keywords:** Integrated Healthcare Alliance, Urban Healthcare Group, Primary Care, Iron Triangle of Healthcare

## Abstract

**Introduction:**

In response to the challenges of inequitable healthcare access and underdeveloped primary care systems, integrated care reforms have emerged as a promising solution. This study examines the effectiveness of one such reform, i.e. the establishment of a cross-regional, tightly integrated healthcare alliance (specifically an urban healthcare group) in the resource-constrained Dapeng District of Shenzhen, China. We assess its impact on service delivery performance through the lens of three core health system objectives: accessibility, quality, and affordability.

**Methods:**

We utilised longitudinal data from 2012 to 2023 and defined seven indicators aligned with the three core health system goals: accessibility, quality, and affordability. Both annual and quarterly data were used, with quarterly data applied to the interrupted time-series analysis. We employed an interrupted time-series analysis (ITSA) to estimate both immediate level changes and subsequent slope changes in the trends. Serial autocorrelation was assessed using the Durbin-Watson (DW) test. Where autocorrelation existed, we used Prais–Winsten generalised least squares (GLS) to ensure robust estimation. Furthermore, we analysed bidirectional referral data to corroborate the effectiveness of the hierarchical diagnosis and treatment system. We also incorporated per-capita medical expenditure, drawn from district administrative records, to track affordability trends over time.

**Results:**

The reform was associated with significant improvements across quality, accessibility, and affordability. The capacity of local healthcare institutions to manage severe and complex conditions was substantially enhanced, reflected in significant post-reform trend increases in both Level III/IV surgeries (*β_3_* = 14.498, p < 0.001) and Type C/D cases (*β_3_* = 51.743, p < 0.001). Accessibility improved notably through expanded bed capacity, with a significant increase in beds per 1,000 population (*β_3_* = 0.031, p < 0.01). Physician and nurse densities showed significant immediate-level increases at the reform point, but their post-reform trend changes were not statistically significant. Affordability also improved, including immediate increases in the proportion of medical insurance patients (*β_2_* = 0.085, p < 0.05) and the proportion of medical income from medical insurance (*β_2_* = 0.113, p < 0.01). Furthermore, both outpatient and inpatient unit costs in Dapeng remained consistently below the Shenzhen citywide average and exhibited slower growth rates. Finally, the hierarchical care system showed strengthened performance, exemplified by a marked increase in the share of consultations delivered by community health centres (CHCs) from 28.13% in 2019 to 55.43% in 2023.

**Conclusion:**

The integrated urban healthcare group model effectively strengthened primary care delivery in a resource-limited setting, achieving concurrent improvements in access, quality, and affordability. However, the gains in human resources were mainly immediate rather than sustained, suggesting that persistent workforce constraints can limit the translation of infrastructure expansion into long-term accessibility improvements. The experience of Dapeng, Shenzhen, China, offers valuable and transferable insights for health systems in comparable contexts seeking to implement integrated care reforms.

## Introduction

Equitable allocation of healthcare resources poses a persistent challenge for health systems worldwide. Fragmented healthcare provision and limited resources often result in the concentration of high-quality medical services in central urban areas, leaving peripheral regions chronically underserved [1,2]. Integrated service delivery models are designed to rectify these resource mismatches by reorganising dispersed healthcare assets around population health needs, thereby facilitating accessible, high-quality, and affordable healthcare services. By minimising duplicate testing and improving operational efficiency, these models seek to optimise resource allocation and enhance overall health system performance [3,4]. Globally, prominent examples include managed care in the United States [5,6], the United Kingdom’s National Health Service (NHS) [7], Singapore’s healthcare group models [8], and Germany’s integrated care programmes (ICPs) [9]. In China, reform efforts have focused on establishing integrated healthcare alliances such as urban healthcare groups and county healthcare communities. These alliances vertically integrate tertiary hospitals with primary care institutions and horizontally link specialised resources to fundamentally reshape care delivery [10].

As a pioneer in China’s healthcare reform, Shenzhen offers instructive insights through its innovative organisational models [11]. In June 2017, it established a cross-regional Dapeng New District Medical and Health Group, a government-funded alliance led by a high-level hospital. The Group employs principles of provider integration including disciplinary alliances, resource sharing, standardised management, and coordinated referrals to strengthen primary care in a remote, underserved area [12]. A tertiary hospital provides clinical and managerial leadership, supported by three district-level sub-hubs (general, maternal and child, and rehabilitation hospitals), which network with community health centres (CHCs) as first-contact points for patients (Figure 1). The Group implements twelve integrated management measures covering administration, human resources, talent development, digital infrastructure, hierarchical referrals, family-doctor contracting, chronic-disease management, integrated diagnostics, pharmacy management, performance evaluation, medical research, and logistics. A unified governance structure consolidates strategy, quality, human resources, finance, and data management, replacing prior fragmentation. Shared clinical and support platforms including standardised care pathways, two-way referrals, telemedicine, diagnostic sharing, and a unified pharmacy system facilitate downward diffusion of expertise and upward referral of complex cases. Family-doctor teams at CHCs coordinate prevention, chronic care, and rehabilitation, optimising resource use, reducing unnecessary referrals, and enhancing continuity of care [13,14]. This framework channels high-quality resources to the community while preserving advanced care at sub-hubs and the core hospital, operationalising tiered service delivery under unified governance.

**Figure 1:**
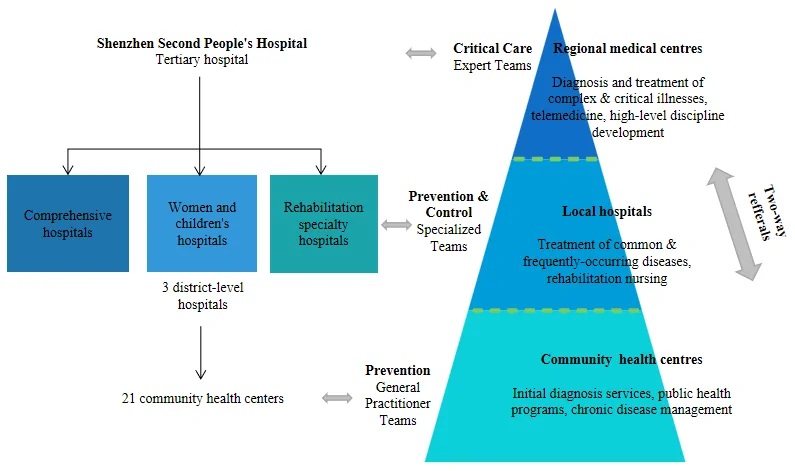
Organisational structure of Shenzhen Dapeng New District Medical and Health Group.

The establishment of integrated healthcare models is often driven by severe regional resource disparities, a challenge that also motivated the creation of the Dapeng Healthcare Group. Although Shenzhen as a whole has 5,431 healthcare institutions, including 32 tertiary hospitals and 926 CHCs, Dapeng District, covering about one-sixth of the city’s land area, was served by only three public hospitals (none tertiary-grade) and 21 CHCs. This imbalance resulted in an estimated 90% of residents seeking medical care outside the district, reflecting broader core-periphery patterns observed in other health systems [15,16,17]. Similar geographic inequities have been documented internationally [18]. In the United States, rural areas continue to face shortages of physicians, hospitals, and specialist services, with mortality rates reported to be approximately 20% higher than in urban areas [19]. Comparable disparities have also been reported in India, where uneven spatial distribution of healthcare resources restricts access in remote and underserved regions [20]. In response to fragmentation and unequal service availability, many countries have adopted integrated care models to strengthen coordination across providers, sectors, and levels of care. Examples include integrated delivery systems such as Kaiser Permanente in the United States [5,6], integrated health and social care in England [7], regional healthcare groups in Singapore [8], and disease management programmes in Germany [9].

Despite the widespread adoption of integrated care systems, their impact on strengthening primary care capacity remains a subject of considerable debate in health policy and services research. Central to this debate are concerns regarding the equity of resource allocation, particularly whether integrated models reduce or inadvertently reinforce inter-regional disparities. Critics argue that integration may entail administrative complexity and coordination costs, potentially reducing efficiency [21]. Proponents, however, suggest that integration fosters synergies such as technology sharing, standardised protocols, and interoperable information systems that can minimise diagnostic errors, reduce service duplication, and enhance primary care quality and efficiency [12,22,23]. By doing so, integration is expected to advance health equity and improve access for underserved populations. Skeptics caution that integrated structures may unintentionally concentrate resources around dominant tertiary hospitals, diverting patients, personnel, and financing away from peripheral facilities. This “hollowing out” of community-based primary services could exacerbate structural inefficiencies and raise system-wide costs [24,25,26,27,28].

Empirical evidence on the effects of integrated care reforms remains mixed, partly due to differences in outcome measures, research design, and conceptual frameworks. Recent reviews suggest that the benefits of integrated and vertically coordinated care vary substantially across contexts, outcomes, and organisational arrangements [29,30]. Some studies report improvements in service coordination, utilisation, and primary care capacity [22,25], while others document limited quality gains, rising costs, or resource concentration around major hospitals [24,27,28]. Evidence on vertical integration further shows that changes in care delivery processes do not necessarily translate into improved quality outcomes [31]. Moreover, much of the literature evaluates integration using single-dimension outcomes such as patient satisfaction, provider perceptions, utilisation, efficiency, or specific clinical indicators rather than considering multiple health-system goals simultaneously [21,32]. Few studies explicitly examine the trade-offs between access, quality, and affordability, leaving it unclear whether gains in one dimension may compromise others [33,34,35]. Consequently, there is a pressing need for comprehensive, multi-dimensional evaluations that assess integrated care models against core objectives of accessibility, quality, and affordability.

To address this gap, this study is grounded in Kissick’s “iron triangle” framework (1994), which conceptualises three interdependent health system goals (access, quality, and cost affordability) and highlights the inherent tensions among them [33]. In this framework, access refers to the timeliness, geographical availability, cultural acceptability, and organisational availability of appropriate services; quality denotes effectiveness, safety, and person-centredness of care; and affordability captures both financial protection for households and fiscal sustainability at the system level. These dimensions often exhibit trade-offs: expanding access through increased capacity or broader insurance coverage may raise higher utilisation and cost pressures; improving quality and safety typically requires investments in skilled workforce, technology, and governance; and tight cost-control can compromise both service availability and care quality. Kissick’s framework was later expanded by the Institute for Healthcare Improvement (IHI) into the “Triple Aim”, which explicitly aims to improve the patient experience, enhance population health, and reduce per-capita costs [34]. Both the “Iron Triangle” and the “Triple Aim” are closely aligned with the World Health Organization (WHO)’s vision of Universal Health Coverage (UHC), which seeks to ensure all people can access quality health services without suffering financial hardship [35,36,37]. By integrating these perspectives, this study provides a comprehensive lens to evaluate whether integrated care models simultaneously advance access, quality, and affordability, thereby addressing a critical gap in the existing literature.

Guided by the access-quality-affordability framework (Figure 2), this study assesses the effectiveness of the tightly integrated Dapeng Healthcare Group in navigating trade-offs to enhance primary care. Our investigation is structured around two central questions. First, did the establishment of the Dapeng Group improve performance across all three facets of the “iron triangle”, or were gains in one area achieved at the expense of others? Second, can a rigorous evaluation of this model provide actionable insights for managing the “iron triangle” in similar resource-constrained settings, such as the peripheries of Chinese city clusters and other low- and middle-income countries? To address these questions, we employ an interrupted time-series analysis (ITSA) to assess how organisational integration affected service accessibility, quality, and affordability. This study makes three contributions. First, it provides a multi-dimensional empirical assessment of a tightly integrated healthcare group, advancing beyond the single-outcome evaluations common in prior research. Second, it applies a longitudinal quasi-experimental design (ITSA) to distinguish immediate level changes from post-reform trends, showing how integration effects evolve over time. Third, it examines a resource-constrained urban periphery, revealing that organisational integration can strengthen local capacity and affordability, while highlighting that sustained improvements in accessibility require complementary workforce and system-level reforms.

**Figure 2:**
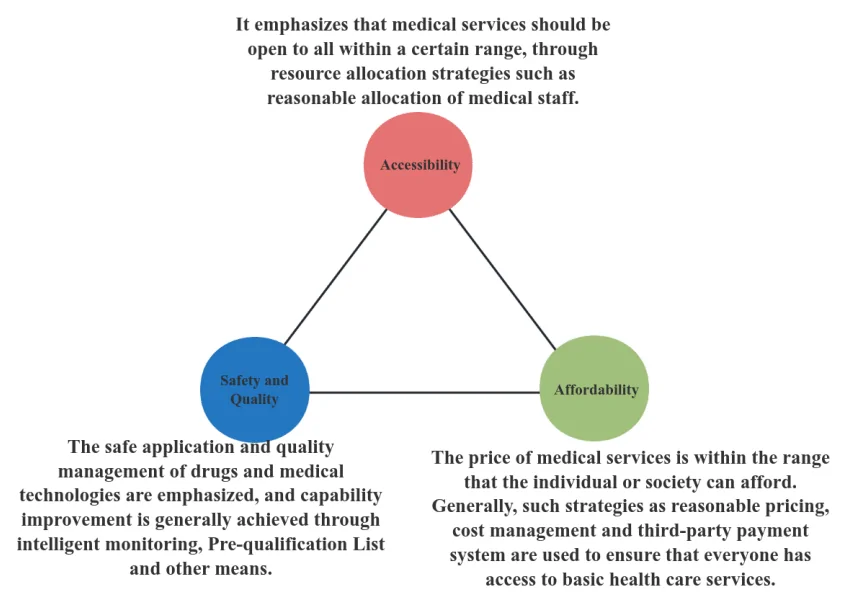
The analytical framework of assessing integrated healthcare model.

## Methods

### Data Sources

Data were primarily sourced from the information platform of the Shenzhen Dapeng New District Medical and Health Group, including both annual and quarterly data covering the period 2012–2023. Key variables included numbers of beds, licensed (and assistant) physicians, registered nurses, Level III/IV surgeries, type C/D cases, numbers of insured patients, and health-insurance revenue. Following prior studies [12,38,39], we selected seven indicators across three core dimensions (Table 1): (1) Accessibility, including the number of Beds per 1,000 population, number of physicians and assistant physicians per 1,000 population, and number of registered nurses per 1,000 population. (2) Quality, measured by the number of level III/IV surgeries and number of type C/D cases. (3) Affordability, represented by the proportion of medical insurance patients and proportion of medical revenue derived from medical insurance funds. Recognising that tiered care is foundational to the urban healthcare group model, we further incorporated internal two-way referral data for 2017–2023 to assess care coordination effects. In addition, annual per-patient medical expenditure, including average cost per outpatient visit and per inpatient admission, was obtained from administrative records of the Shenzhen Healthcare Security Administration. These figures were used to evaluate affordability trends and compare Dapeng’s performance against the Shenzhen citywide average.

**Table 1:** Evaluation Indicators and Calculation Formula.


DIMENSION	EVALUATION INDICATOR	CALCULATION FORMULA

Accessibility	Number of beds per 1,000 population	(Number of Beds in Medical Institutions/Resident Population) × 1,000

	Number of physicians and physician assistants per 1,000 population	(Number of Physicians + Assistant Physicians)/Resident Population × 1,000

	Number of registered nurses per 1,000 population	Number of Registered Nurses in Medical Institutions/Resident Population × 1,000

Quality	Number of level III/IV surgeries	Number of Level III Surgeries + Number of Level IV Surgeries

	Number of type C/D cases	Number of Type C Cases + Number of Type D Cases

Affordability	Proportion of medical insurance patients	Number of Patients Reimbursed by medical insurance/Total Number of Patients

	Proportion of medical revenue from medical insurance	Medical insurance fund revenue/medical revenue


*Note:* Surgical procedures are classified into four levels (I–IV) based on risk and complexity; level III/IV surgeries denote those that are relatively complex, technically demanding, and high-risk. Cases are categorised into four types (A-D) according to illness severity; type C/D cases represent conditions that are complex and present high diagnostic difficulty.

### Research Design

ITSA is a robust quasi-experimental method widely used to evaluate the impact of health policy interventions when randomised controlled trials (RCTs) are not feasible. By leveraging longitudinal data collected at multiple time points before and after an intervention, ITSA fits a segmented linear regression model to estimate both immediate changes in level and sustained changes in trend following the policy implementation. First formalised by Box and Tiao (1975), this approach has been extensively applied in health policy evaluation [40,41]. In this study, we adopted a single-group ITSA design using the pre-intervention period as the control to assess changes in the level and trend of key outcome indicators following the establishment of the urban healthcare group. The segmented regression model is specified as follows:


$$Y_{t}=\beta_{0}+\beta_{1}X_{1}+\beta_{2}X_{2}+\beta_{3}X_{3}+\varepsilon$$

The intervention point (reform implementation) was set at the second quarter of 2017 (2017Q2), when the Dapeng Healthcare Group was formally established. Where *Y_t_* is the indicator at time *t*; *X_1_* is a time variable; *X_2_* is a binary intervention indicator (0 pre-reform, 1 post-reform); *X_3_* is a post-intervention time variable (0 pre-reform; thereafter, sequence of post-reform points). *β_0_* is the baseline level; *β_1_* is the pre-reform slope; *β_2_* is the immediate level change at reform; *β_3_* is the change in slope post-reform; and ɛ is the error term. This model allowed for the assessment of both immediate and long-term effects of the reform on accessibility, quality, and affordability within local healthcare institutions. To account for potential serial autocorrelation, we employed the Durbin-Watson (DW) test. A DW statistic close to 2 suggests independence of errors. Where significant autocorrelation was detected, parameter estimates were adjusted using generalised least squares estimation via the Prais-Winsten procedure to ensure robustness. All analyses were performed using Stata 18.0, with a two-sided significance level of α = 0.05.

This study utilised secondary administrative data and did not directly involve patients or the public in study design, data collection, or analysis. While their perspectives are valuable for understanding integrated care experiences, the retrospective and macro-level nature of this analysis limited such involvement. Future studies should incorporate patient-reported outcomes and participatory methods to complement these findings.

## Results

### Accessibility

Following the establishment of the integrated healthcare alliance, accessibility of local primary care improved, mainly driven by the redistribution of bed resources to the primary level and the implementation of a tiered diagnosis and treatment system (Table 2, Figure 3). Between 2012 and 2023, most accessibility indicators increased over time, although sustained post-reform trend improvements were observed only for bed availability. Specifically, bed availability showed a significant post-reform trend increase (*β_3_* = 0.031, p < 0.01), indicating that the reform contributed to a lasting expansion in local inpatient capacity. This enhancement enabled primary care institutions to accommodate more patients locally, reducing the need for residents to seek inpatient services outside the district.

**Table 2:** Impact of the reform on the service performance of primary care institutions.


DIMENSION	INDICATOR	MODEL	*N*	*β_0_*	*β_1_*	*β_2_*	*β_3_*	*DW*	*AR(1)ρ*	*R^2^*

Accessibility	Number of beds per 1,000 population	Prais-Winsten	48	1.807*** (0.083)	0.024** (0.006)	0.186 (0.095)	0.031** (0.008)	1.955	0.451	0.925

	Number of physicians and physician assistants per 1,000 population	OLS	48	1.290*** (0.098)	0.011 (0.008)	0.386** (0.125)	–0.006 (0.009)	1.653	—	0.648

	Number of registered nurses per 1,000 population	OLS	48	1.383*** (0.059)	0.021** (0.005)	0.152* (0.075)	–0.006 (0.006)	1.757	—	0.861

Quality	Number of level III/IV surgeries	OLS	48	–3.803 (31.279)	0.989 (2.491)	3.768 (39.96)	14.498*** (3.020)	2.080	—	0.829

	Number of type C/D cases	Prais-Winsten	48	234.760* (103.363)	6.862 (7.815)	125.046 (110.134)	51.743*** (10.430)	1.940	0.344	0.863

Affordability	Proportion of medical insurance patients	OLS	48	0.290*** (0.028)	0.010** (0.002)	0.085* (0.035)	–0.002 (0.003)	2.220	—	0.883

	Proportion of medical income from medical insurance	OLS	48	0.408*** (0.026)	0.005 (0.002)	0.113** (0.033)	–0.001 (0.003)	2.360	—	0.803


*Notes:* Standard errors are shown in parentheses. *p < 0.05, **p < 0.01, ***p < 0.001. Ordinary Least Squares (OLS) was used when the DW statistic was close to 2 (indicating no significant serial autocorrelation). Prais-Winsten generalised least squares with AR(1) correction was applied when the DW statistic deviated substantially from 2, to account for first-order autocorrelation. AR(1)*ρ*: first-order autocorrelation coefficient; values near 0 indicate weak autocorrelation. For OLS models, “—” indicates not applicable. For Prais-Winsten models, DW is the transformed value.

**Figure 3:**
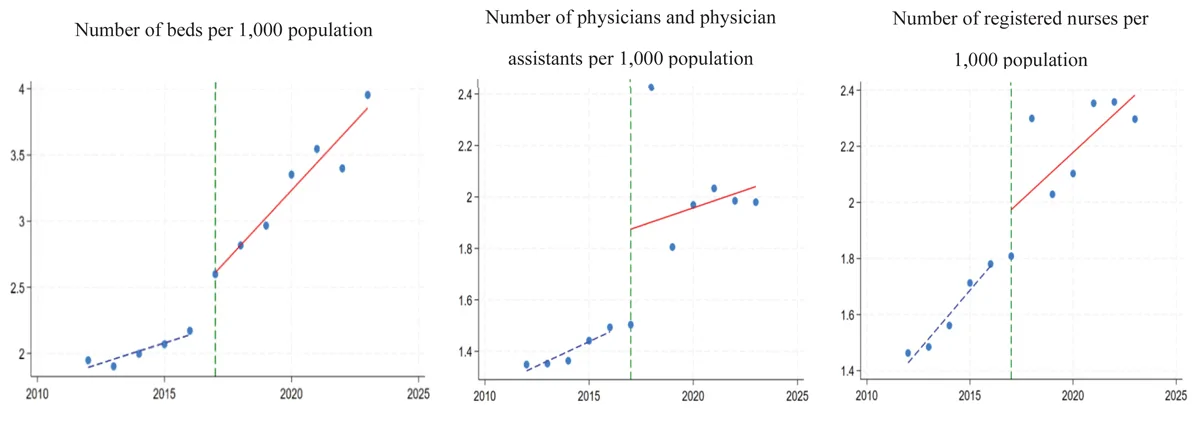
Trends in accessibility evaluation indicators before and after reform.

Regarding human resources, the density of physicians and assistant physicians exhibited a significant immediate increase at the intervention point (*β_2_* = 0.386, p < 0.01), though no significant post-reform trend change was observed. Registered nurse density also increased modestly immediately following the reform (*β_2_* = 0.152, p < 0.05), but its post-reform trend change was not statistically significant. These results suggest that while the reform improved short-term availability of medical and nursing care, persistent workforce constraints limited the conversion of expanded capacity into sustained accessibility gains.

Tiered care delivery further strengthened local access to primary services, reflected in the enhanced first-contact system at the community level and the effective operation of the two-way referral mechanism. Specifically, the proportion of outpatient visits handled by CHCs increased substantially from 28.13% in 2019 to 55.43% in 2023 (Table 3). This shift suggests that more residents were able to access basic medical services close to home, reducing dependence on higher-level hospitals and supporting the policy goal of managing common and frequently occurring conditions at the primary care level.

**Table 3:** Two-way referral volumes and CHCs outpatient share within the Healthcare Group.


YEAR	TERTIARY ⇋ DISTRICT HOSPITALS		DISTRICT HOSPITALS ⇋ CHCS		THE PROPORTION OF OUTPATIENT VISITS AT CHCS
	
	DOWN	UP	DOWN	UP	

2017	115	105	1154	7951	–

2018	301	282	1282	11000	–

2019	584	306	1374	17307	28.13%

2020	205	461	1954	12248	26.96%

2021	234	341	3877	26721	39.76%

2022	206	347	1583	28580	57.29%

2023	170	240	7480	23513	55.43%

Overall	1815	2082	18704	127320	–


*Notes:* DOWN: Downward referrals; UP: Upward referrals.

### Quality

The reform was associated with a substantial strengthening of local capacity to manage complex and severe conditions. Notably, there was a gradual increase in the ability of primary-level institutions within the integrated group to perform Level III/IV surgeries. While the number of such surgeries did not show a significant immediate change at the intervention point (*β_2_* = 3.768, p > 0.05), the post-reform trend increased significantly *(β_3_* = 14.498, p < 0.001). This shift of advanced surgical procedures to primary-level institutions (mainly level-one hospitals) was facilitated by targeted technology diffusion, skill transfer, and standardised training, collectively enhancing the technical capacity and service quality required for complex interventions, and thereby elevating overall primary care standards.

Similarly, improvements were observed in the management of type C/D cases. After applying the Prais–Winsten AR(1) correction, no significant immediate level change was detected (*β_2_* = 125.046, p > 0.05), but the post-reform trend increased significantly (*β_3_* = 51.743, p < 0.001). These findings indicate enhanced diagnostic and therapeutic capabilities for severe conditions within primary care settings. The consistent upward trends in both metrics indicate that the reform contributed to a gradual, sustained strengthening of local capacity to manage complex procedures and high-acuity cases (Table 2, Figure 4).

**Figure 4:**
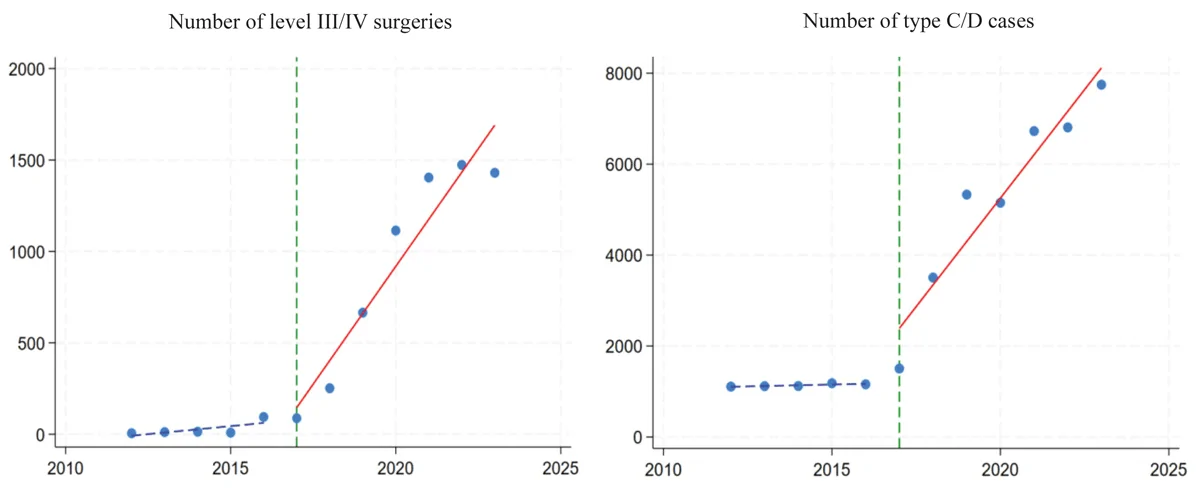
Trends in quality evaluation indicators before and after reform.

### Affordability

The reform was associated with improvements in healthcare affordability. From 2012 to 2023, the proportion of medical insurance patients among all patients treated within the district increased over time, with a significant immediate increase following the reform (*β_2_* = 0.085, p < 0.05), while the post-reform trend change was not statistically significant. This suggests that the reform was associated with an immediate increase in the share of insured patients using local healthcare services, followed by a relatively stable trend. Similarly, the proportion of medical revenue derived from medical insurance increased significantly at the intervention point (*β_2_* = 0.113, p < 0.01), with no significant post-reform trend change (Table 2, Figure 5). Considering that Shenzhen had achieved universal insurance coverage by 2007, these post-reform increases suggest that more insurance-financed care was retained within the district and that the financial sustainability of local institutions improved.

**Figure 5:**
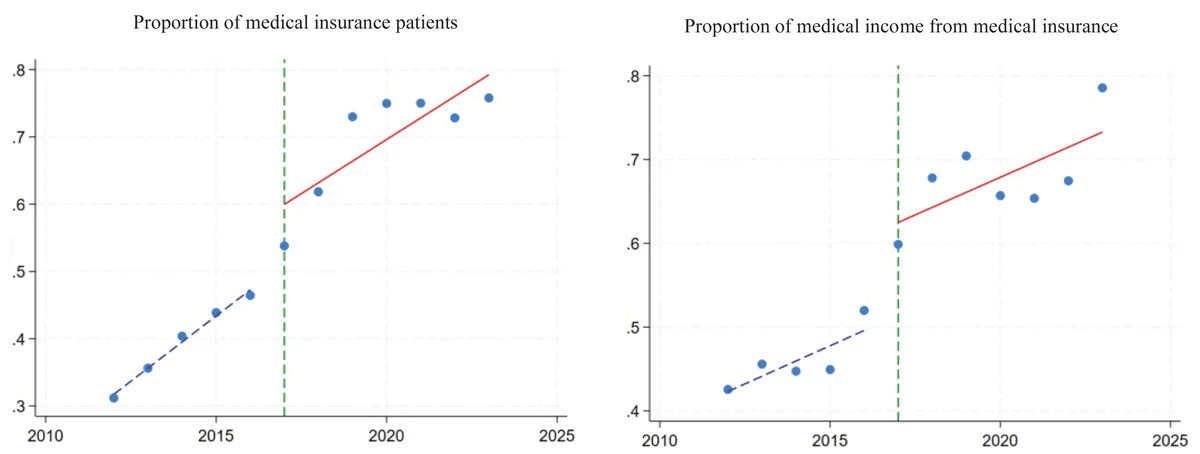
Trends in affordability evaluation indicators before and after reform.

Administrative spending data further confirm these affordability gains (Figure 6). From 2012 to 2023, both outpatient and inpatient unit costs in Dapeng remained consistently below the Shenzhen citywide averages by approximately 34.4% for outpatient visits and 20.3% for inpatient admissions. This cost differential widened over time. The outpatient gap increased from 18.3% lower in 2012 ($19.6 vs $23.9) to 56.0% lower in 2023 ($24.0 vs $54.6), while the inpatient gap increased from 18.0% ($778.2 vs $949.1) to 25.1% ($1,565.2 vs $2,089.1). Moreover, cost growth in Dapeng was more moderate, with compound annual growth rates (CAGRs) of 1.90% for outpatient and 6.56% for inpatient costs, compared to citywide rates of 7.79% and 7.44%, respectively. Notably, outpatient unit cost in Dapeng peaked in 2020 and declined through 2023. These findings are consistent with sustained improvements in affordability, accompanied by greater insurance-financed local service use and stricter cost containment. These factors resulted in lower and more stable medical expenditures for residents in Dapeng compared to those for the broader Shenzhen population.

**Figure 6:**
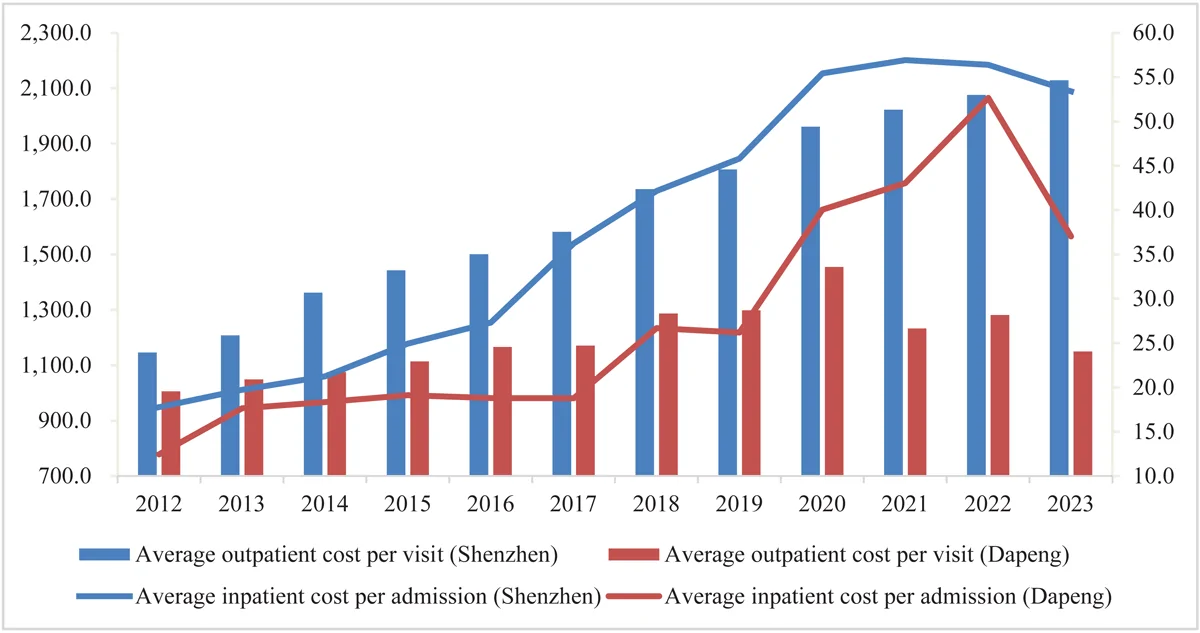
Average outpatient cost per visit and inpatient cost per admission in Shenzhen and Dapeng (2012–2023). *Notes:* The Consumer Price Index (CPI) varied minimally over the period (index 100.2–102.9), so values are shown in nominal terms without inflation adjustment. Costs are presented in US dollars (US$), converted from RMB at the period-average exchange rate of US$1 = 6.5757 (2012–2023) [42].

### Robustness and Model Diagnostics

To assess model validity and robustness, we conducted a series of diagnostic and sensitivity analyses consistent with the final ITSA specifications. Serial autocorrelation was assessed using the Durbin–Watson statistic and residual autocorrelation function (ACF) plots. Models exhibiting autocorrelation were re-estimated using the Prais–Winsten AR(1) correction, with adjusted Durbin–Watson statistics and AR(1) parameters reported. Additional diagnostics included residual-versus-fitted plots and normal Q-Q plots. Heteroskedasticity was evaluated using the Breusch–Pagan/Cook–Weisberg and White tests. For models showing evidence of heteroskedasticity, Newey–West heteroskedasticity- and autocorrelation-consistent (HAC) standard errors were estimated.

A placebo test was further performed by assigning 2014Q4 as a false pre-intervention point. This period was selected to minimise potential contamination from subsequent policy developments, including the national promotion of hierarchical diagnosis and treatment in 2015, Shenzhen’s citywide expansion of primary healthcare group reforms in 2016, and early specialist-alliance arrangements before the formal establishment of the Dapeng Healthcare Group in 2017. Across all diagnostic and robustness analyses, the main findings remained substantively consistent. Full model outputs, diagnostic plots, heteroskedasticity tests, Newey-West HAC estimates, and placebo test results are provided in the Supplementary Materials.

## Discussion

This study advances integrated care research by examining how organisational integration relates to the three objectives of the healthcare “iron triangle” (i.e., access, quality, and affordability). Drawing on longitudinal quasi-experimental evidence from a resource-constrained urban periphery, the findings suggest that a tightly integrated urban healthcare group can strengthen local service capacity and improve affordability outcomes. Importantly, the study indicates that sustained accessibility gains are limited when workforce integration does not keep pace with infrastructure expansion.

Consistent with prior research, tight vertical integration between hospitals and primary care institutions can increase local service utilisation and support care shifts towards lower-level facilities [11,12,22,43]. Complementary reforms, including family-doctor contracting and enhanced referral mechanisms, have been shown to improve spatial equity and strengthen first-contact access [23]. International evidence also suggests that integrated community-based models can improve timely access, reduce emergency visits and acute admissions, and expand preventive service use among disadvantaged populations [44,45]. In line with these findings, the Dapeng case achieved partial accessibility gains, including increased bed availability and a larger share of outpatient visits delivered by CHCs. This indicates that residents were increasingly able to obtain basic care locally.

However, persistent workforce constraints limited the translation of infrastructure expansion into full accessibility improvements. Despite a post-reform doubling of bed resources, physician and nurse densities did not increase proportionally, restricting reductions in waiting times and first-contact delays. This aligns with broader literature noting resource coordination challenges in integrated systems [11,41,46,47,48], and highlights structural barriers such as rigid staffing quotas and the limited attractiveness of primary care careers. Under locality-based personnel management and weak career advancement pathways, primary care institutions struggle to counteract the “siphoning effect” of urban tertiary hospitals [49]. These findings underscore that workforce reform should be a central component of integrated care initiatives rather than a secondary consideration.

Quality indicators also improved under the Dapeng model. Significant post-reform increases in Level III/IV surgeries and type C/D cases suggest enhanced local capacity to manage complex and severe conditions. These findings are consistent with evidence that primary-care-centred integration can enhance perceived care quality [13] and with national assessments emphasising workforce capability and clinical governance as prerequisites for quality improvement [11]. Unlike some studies reporting integration-driven cost increase without quality gains [24,28], the Dapeng model combined clinical integration (e.g., standardised pathways, two-way referrals, telemedicine, shared diagnostics) with managerial alignment (e.g., unified governance, value-oriented performance appraisal), facilitating expertise diffusion and reducing practice variation [12]. These results extend the literature by showing that tightly integrated delivery systems can strengthen complex-case capability at the primary level under unified governance, without the cost escalation reported in some consolidation settings.

Affordability-related outcomes also improved. While integrated, primary-care-centred models can reduce service duplication and moderate expenditure growth, these effects are context-dependent [50,51,52]. In Dapeng, outpatient and inpatient unit costs grew more slowly than the municipal average, and the proportion of medical revenue derived from insurance increased and remained high, reflecting stronger financial protection and greater within-district care utilisation. This is noteworthy in the context of near-universal insurance coverage in China, where coverage alone does not guarantee appropriate use of primary care [53,54,55,56,57,58]. The findings suggest that integrated reforms can both improve financial indicators and influence patient care-seeking behaviour, supporting more appropriate and efficient use of local services.

The Dapeng experience also provides insight into the potential to mitigate the conventional trade-offs of the “iron triangle”, which posits inherent tensions among access, quality, and cost control [37,59]. In Dapeng, concurrent gains were observed across all three dimensions. Quality improved through advanced surgical and complex-case capabilities; affordability was enhanced through increased insurance coverage and moderated cost growth; and accessibility advanced through expanded bed capacity and a greater share of care delivery locally. This pattern is consistent with research suggesting that organisational integration can reduce unwarranted variation and disseminate advanced capabilities across networks [11,22,23]. It stands in contrast, however, to studies linking integration solely to higher costs without quality improvements [24,28]. The divergence can be attributed to Dapeng’s governance and operational innovations such as unified management, standardised clinical pathways, interoperable information systems, and value-oriented purchasing incentives. These mechanisms contained costs, reduced inefficiencies, and shifted the system’s efficiency frontier outward.

The Dapeng case demonstrates that, under specific structural and managerial conditions, the classic trade-offs of the “iron triangle” can be partially mitigated. By strategically aligning resources, incentives, and clinical processes, tightly integrated healthcare models can help improve access, quality, and affordability simultaneously under supportive institutional conditions. For practitioners seeking to replicate or adapt such models, certain institutional features of the Dapeng experience are likely to be broadly transferable (Table 4). These include technical and managerial mechanisms such as vertical integration across care levels, standardised referral pathways, interoperable diagnostic and information platforms, family-doctor coordination, and integrated performance management. These mechanisms primarily involve organisational design and operational alignment, making them adaptable across diverse healthcare systems. In contrast, other institutional arrangements are highly context-specific and may be difficult to replicate without similar governance and fiscal conditions. These include three-level government–hospital collaboration, unified legal representation, separation of ownership and operating rights, and sustained fiscal support. The feasibility and effectiveness of these features depend on local administrative capacity, public hospital governance structures, and political commitment to long-term investment in integrated care. While technical and managerial innovations can be adapted in other urban healthcare contexts, successful replication requires careful consideration of the enabling governance and fiscal environment. Practitioners should prioritise transferable mechanisms to achieve tangible improvements in access, quality, and affordability, while acknowledging the constraints imposed by context-specific institutional arrangements.

**Table 4:** Transferability of key institutional features of the Dapeng model.


INSTITUTIONAL FEATURE	ROLE IN THE DAPENG MODEL	TRANSFERABILITY	KEY CONDITION FOR REPLICATION

Vertical integration of municipal hospitals, district hospitals, and CHCs	Links advanced care, district-level services, and first-contact primary care	High	Existence of a referral network and willing lead hospital

Unified governance structure with a shared legal representative and group leadership	Reduces fragmentation, clarifies accountability, and strengthens coordination across member institutions	Moderate	Requires compatible public hospital governance rules and strong administrative coordination

Standardised two-way referral pathways	Supports tiered care and continuity between CHCs and hospitals	High	Shared referral standards and reserved referral capacity

Shared diagnostic, pharmacy, and information platforms	Improves resource sharing and reduces duplication	High	Digital infrastructure and interoperable information systems

Integrated performance management	Aligns incentives across member institutions	Moderate	Requires comparable indicators and enforceable evaluation rules

Family-doctor teams and CHC gatekeeping	Strengthens first-contact care and chronic disease management	High	Sufficient primary care workforce and community trust

Three-level government–hospital collaboration	Provides political support and cross-level coordination	Context-specific	Depends on local government capacity and intergovernmental cooperation

Separation of ownership and operating rights	Allows district institutions to retain ownership while integrating operations	Context-specific	Depends on local public asset and hospital management regulations

“Four unchanged” principle, including affiliation, personnel identity, asset ownership, and fiscal input	Reduces resistance during integration	Context-specific	Requires stable public-sector employment and fiscal arrangements

Local fiscal support and appointed financial oversight	Supports implementation and financial accountability	Context-specific	Depends on fiscal capacity and public financing mechanisms

Staffing and talent development arrangements	Supports technical diffusion but remains a bottleneck	Moderate to low	Requires workforce mobility, incentives, and career pathways


Based on the Dapeng case study, a set of integrated policy recommendations for improving primary care in resource-constrained urban areas can be articulated as follows. First, policymakers should promote vertical integration across different healthcare providers to create a unified governance and operational network, enhancing access, strengthening tiered referrals, and enabling the diffusion of clinical expertise to lower-level facilities. This should be complemented by standardised two-way referral pathways and shared digital platforms. Second, sustained accessibility and quality require investments in primary care workforce development, including recruitment, training, and the establishment of family-doctor teams to manage chronic conditions and first-contact services effectively. Finally, financial oversight and incentive alignment through local fiscal support, appointed financial managers, and value-oriented performance appraisal can moderate cost growth, increase affordability, and encourage appropriate utilisation of local services.

This study has several limitations. First, although ITSA is suitable for evaluating population-level policy interventions, the single-group design lacked a contemporaneous control area. Consequently, the findings should be interpreted as reform-associated changes rather than definitive causal effects, as concurrent policies, secular trends, or external shocks such as the COVID-19 pandemic may have influenced healthcare utilisation. Future research should adopt comparative or multi-site designs with contemporaneous control groups to strengthen causal inference and isolate the effects of integrated care reforms. Second, reliance on aggregate administrative data limited adjustment for patient-level factors, including case mix, disease severity, and socioeconomic characteristics, and precluded formal heterogeneity analyses across facility types or service categories. Future studies should incorporate facility-level and patient-level data to capture heterogeneity in outcomes and identify which populations or institutions benefit most from integration. Finally, the available data did not permit formal mechanism analysis. Although the discussion identifies plausible pathways, including referral coordination, workforce deployment, local service capacity, care-seeking behaviour, and insurance-financed care, consistent process-level indicators were unavailable. Future studies should use such data to examine how integration operates through these mechanisms to influence access, quality, and affordability.

## Conclusion

The study suggests that tightly integrated urban healthcare groups can help strengthen primary care in resource-constrained urban regions. Over time, the reform was associated with improved local service capacity, greater use of community-based care, and enhanced affordability-related indicators, although persistent workforce constraints limited the extent of sustained accessibility gains. These findings indicate that the trade-offs implied by the healthcare “iron triangle” may be moderated when organisational integration is supported by aligned governance, operational coordination, and incentive arrangements. Beyond the substantive findings, this study has both theoretical and practical implications. Theoretically, it contributes to the integrated care literature by providing a multi-dimensional evaluation of organisational integration through the combined lens of access, quality, and affordability. Practically, the study highlights that the effectiveness of integrated healthcare alliances depends not only on structural integration but also on the alignment of governance, operational coordination, and incentive arrangements. While not a universally applicable blueprint, the experience offers a set of adaptable institutional and managerial approaches that can inform integrated care reforms in other underserved or resource-constrained urban settings.

## Additional File

The additional file for this article can be found as follows:

10.5334/ijic.10192.s1 Supplementary Materials. Model Diagnostics, Heteroskedasticity, and Placebo Tests for Interrupted Time Series Analysis.
