# Supplementary material for: The “Iron Triangle” of Healthcare Services: Evaluating an Integrated Healthcare Alliance’s Impact on Primary Care in Shenzhen, China
Source: Int J Integr Care. 2026 Sep 30;26(3):22. doi: 10.5334/ijic.10192 (PMC13635666; doi:10.5334/ijic.10192)
Supplement: Supplementary file 1 [file ijic-26-3-10192-s1.pdf]

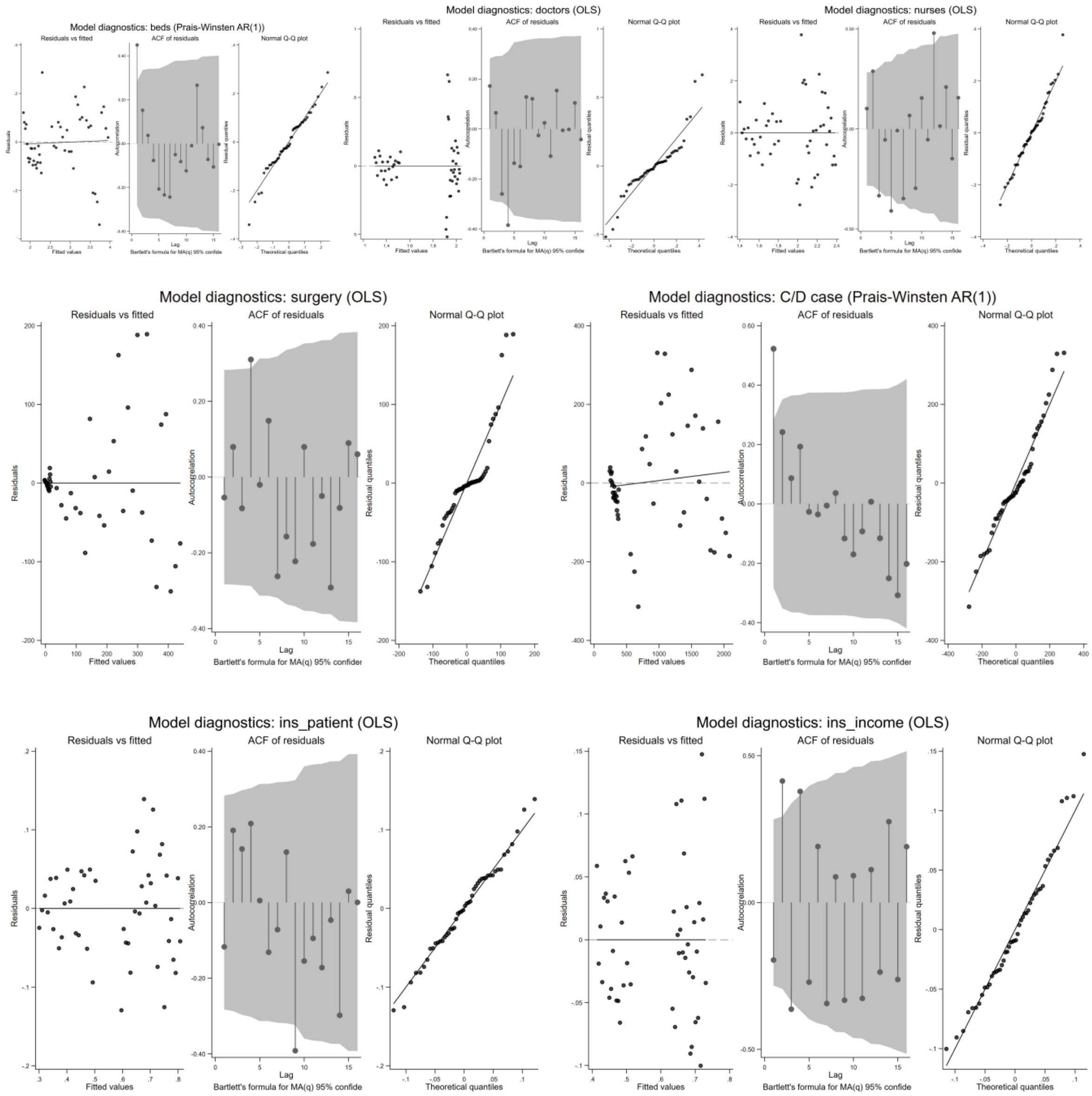

Supplementary Figure 1 Model diagnostic plots

Notes: Diagnostic plots include residual-versus-fitted plots, residual ACF plots, and normal Q-Q plots for each ITSA model. Overall, the plots did not suggest severe model misspecification. Mild residual dependence or distributional departures in selected outcomes were addressed using Prais–Winsten correction and Newey–West HAC robustness checks.

Supplementary Table 1 Heteroskedasticity diagnostics for the ITSA models

DIMENSION	INDICATOR	*N*	BP/Cook-Weisberg χ^2	*P value*	White χ^2	*P value*
Accessibility	Number of beds per 1000 population	48	8.915	0.003	14.602	0.012
	Number of physicians and physician assistants per 1000 population	48	10.165	0.001	29.460	0.001
	Number of registered nurses per 1000 population	48	3.515	0.061	20.910	0.001
Quality	Number of level III/IV surgeries	48	32.182	0.001	17.824	0.003

DIMENSION	INDICATOR	*N*	BP/Cook-Weisberg χ^2	*P value*	White χ^2	*P value*
Affordability	Number of type C/D cases	48	5.766	0.016	18.597	0.002
	Proportion of medical insurance patients	48	4.886	0.027	7.532	0.184
	Proportion of medical income from medical insurance	48	4.633	0.031	7.647	0.177

Notes: BP/Cook–Weisberg = Breusch–Pagan/Cook–Weisberg test. Evidence of heteroskedasticity was considered present when either the BP/Cook–Weisberg test or the White test was statistically significant at $p < 0.05$. Partial evidence indicates that only one of the two tests was statistically significant. Because several models showed evidence of heteroskedasticity, Newey–West HAC estimates were used as a robustness check.

Supplementary Table 2 Newey-West HAC robustness estimates

DIMENSION	INDICATOR	*N*	β_0	β_1	β_2	β_3
Accessibility	Number of beds per 1000 population	48	1.823***<br>(0.056)	0.020***<br>(0.005)	0.290***<br>(0.072)	0.031***<br>(0.006)
	Number of physicians and physician assistants per 1000 population	48	1.290***<br>(0.031)	0.011***<br>(0.002)	0.386*<br>(0.196)	-0.006<br>(0.009)
	Number of registered nurses per 1000 population	48	1.383***<br>(0.030)	0.021***<br>(0.002)	0.152<br>(0.096)	-0.006<br>(0.005)
Quality	Number of level III/IV surgeries	48	-3.803<br>(2.573)	0.989**<br>(0.300)	3.768<br>(27.217)	14.498***<br>(2.238)
	Number of type C/D cases	48	266.984***<br>(7.233)	1.915**<br>(0.604)	243.878*<br>(130.129)	55.046***<br>(6.626)
Affordability	Proportion of medical insurance patients	48	0.291***<br>(0.008)	0.010***<br>(0.001)	0.085**<br>(0.040)	-0.002<br>(0.002)
	Proportion of medical income from medical insurance	48	0.409***<br>(0.013)	0.005***<br>(0.001)	0.113***<br>(0.031)	-0.001<br>(0.002)

Notes: Standard errors are shown in parentheses. *$p < 0.05$, **$p < 0.01$, ***$p < 0.001$. Newey–West HAC standard errors account for both heteroskedasticity and autocorrelation. The HAC estimates were broadly consistent with the primary ITSA results reported in Table 2 of the main text, particularly for the post-reform trend increases in beds per 1,000 population, Level III/IV surgeries, and Type C/D cases, as well as the immediate increases in insurance-related affordability indicators.

Supplementary Table 3 Placebo test using 2014Q4 as a false intervention point

DIMENSION	INDICATOR	FINAL MODEL	*N*	β_2	β_3	*DW*	*AR(1)ρ*	R^2
Accessibility	Number of beds per 1000 population	Prais-Winsten	48	-0.049<br>(0.105)	0.051<br>(0.034)	1.885	0.500	0.917
	Number of physicians and physician assistants per 1000 population	Prais-Winsten	48	0.079<br>(0.204)	0.007<br>(0.040)	2.157	0.381	0.655
	Number of registered nurses	OLS	48	0.105	0.009	1.578	-	0.848

DIMENSION	INDICATOR	FINAL MODEL	*N*	β_2	β_3	*DW*	*AR(1)ρ*	R^2
Quality	per 1000 population			(0.090)	(0.013)			
	Number of level III/IV surgeries	OLS	48	-79.401 (47.520)	13.021 (6.993)	1.921	-	0.813
	Number of type C/D cases	Prais-Winsten	48	-128.162 (127.150)	68.833 (37.255)	1.972	0.640	0.789
Affordability	Proportion of medical insurance patients	OLS	48	0.015 (0.043)	0.001 (0.006)	1.820	-	0.865
	Proportion of medical income from medical insurance	OLS	48	0.020 (0.043)	0.005 (0.006)	1.798	-	0.749

Notes: Standard errors are shown in parentheses. *p < 0.05, **p < 0.01, ***p < 0.001. Placebo β_2 and β_3 refer to the level and trend changes estimated using 2014Q4 as a pre-intervention false intervention point. DW reports the final-model Durbin–Watson statistic; for Prais–Winsten models, the adjusted DW statistic is reported. The placebo test did not indicate systematic placebo effects across outcomes, supporting the specificity of the reform-associated changes reported in Table 2 of the main text.
